# The Mediating Role of Goal Orientation (Task) in the Relationship between Engagement and Academic Self-Concept in Students

**DOI:** 10.3390/ijerph17228323

**Published:** 2020-11-11

**Authors:** Pablo Usán Supervía, Carlos Salavera Bordás, Víctor Murillo Lorente

**Affiliations:** 1 Departament of Psychology, Faculty of Human Sciences and Education, University of Zaragoza, Valentín Carderera n^o^4, 22003 Huesca, Spain; 2 Departament of Psychology, Faculty of Education, University of Zaragoza, Pedro Cerbuna n^o^12, 50009 Zaragoza, Spain; salavera@unizar.es; 3 Departament of Physiatry and Nursing, Faculty of Health and Sports Sciences, University of Zaragoza, Plaza Universidad n^o^3, 22002 Huesca, Spain; vmurillo@unizar.es

**Keywords:** academic engagement, goal orientation (task), academic self-concept, students, adolescents

## Abstract

Some students many not possess the necessary strategies and skills to meet the demands of academic life and develop negative attitudes, physical and mental exhaustion, and other attitudes that will undermine their personal and academic development. This study analyses the relationship and possible role of goal orientation as a mediator between engagement and academic self-concept. *Methods*: The study concerned a population of 1756 subjects from 12 secondary schools (ESO). The instruments used included the Utrecht Work Engagement Scale—Student (UWES-S), the Perception of Success Questionnaire (POSQ), and the Academic Self-Concept Scale (ASCS). *Results*: The results revealed significant correlations between academic engagement, task-oriented goal orientation, and academic self-concept. In addition, task orientation was found to play a positive mediating role between academic engagement and academic self-concept, leading to adaptive models in secondary school students. *Conclusion*: These results highlight the need to promote goal orientation in order to stimulate self-determined behaviours in the school environment and improved levels of academic self-concept, which in turn will facilitate the psychological and personal development of the student and increase the chances of academic success.

## 1. Introduction

At school, students are exposed to numerous contextual and personal circumstances that may significantly affect their personal and academic development, especially in secondary school, during adolescence, which is a key stage in the life of a person, preceding adulthood [1]. Some students may not possess the necessary strategies and skills to meet the demands of academic life, and they may develop negative attitudes towards themselves and lose motivation, leading to poor academic performance and may ultimately lead to dropping out of school [2,3,4].

One of the most important psychological variables in this regard is academic engagement, a concept derived from positive psychology, which is defined as a psychological state characterised by resilience against stressful and exhausting situations [5]. From an adaptive perspective, academic engagement is characterised by vigour, dedication, and absorption. Vigour denotes energetic and resilient attitudes towards academic demands, the willingness to invest effort, and the ability to overcome obstacles; dedication denotes involvement and commitment to academic task, as well as feelings of enthusiasm and focus; and absorption denotes the ability to engage with a task in ways that are satisfactory and meet the student’s expectations [6].

As such, academic engagement responds to adaptive behaviours characterised by engagement, satisfaction, and commitment to academic tasks. Previous studies have linked academic engagement with satisfactory academic performance [7], high levels of self-perception and self-efficacy [8], low levels of school dropout [9], and more broadly, academic happiness and student wellbeing [10]. Therefore, academic engagement plays a key role in student life, especially during childhood and adolescence, which are key stages for the configuration of adult personality [11].

On the other hand, concerning academic goals, one of the most important socio-cognitive theories is goal orientation theory [12], a frame of reference that is widely used in the field of educational psychology to characterise the motivations, purpose, and intentions that guide student behaviour in academic settings. In achievement environments, the student’s primary goal is to demonstrate skills and capabilities. Two states of motivational commitment can be distinguished: Task-oriented, which is more self-determined, and ego-oriented, which is less self-determined. Task-oriented students tend to believe that academic success results from effort, motivation, and abnegation, whereas ego-oriented students try to demonstrate that their ability and competence is above that of their peers while neglecting some aspects of the former [13]. With two states of motivational commitment distinguished, we measure and focus on task orientation in our research as a more self-determined variable [12].

Specifically, task orientation is defined as an integrated model of beliefs or pattern of attributions that directs the behavioural intentions of a person, formed by different modes of approach, commitment, affection, and response to achievement activities [14]. In this kind of orientation there is more self-determination when students seek learning goals, expressing interest in enriching their knowledge and in developing a new intrinsic ability, in contrast to the meta-ego orientations, which influence motivation, involvement in the task, as well as in the results obtained at the end of it [15].

In this way, numerous studies have found positive correlations between task orientation and variables such as persistence, will, and commitment [16]; motivation in carrying out academic tasks [17]; effort and constancy [18]; academic enjoyment and happiness [19]; the development of better coping strategies [20]; as well as greater levels of physical, psychological, and emotional wellbeing in a school environment among adolescents [21]. Conversely, ego orientation has been related to extrinsic and non-adaptive motivations [22]; cheating and lack of commitment [23] and less psychic and emotional wellbeing [16]. Goal orientation theory thus interprets whether students are task- or ego-oriented based on how they interpret, respond to, and deal with different school achievements [12].

Finally, academic self-concept is an important construct that plays a key role in academic performance [24]. It is defined as a relatively stable psychological construct constituted by a student’s self-perception of academic behaviour and aptitudes [25]. Academic self-concept is one factor of self-perception in general [26] and is closely related to general well-being and individual self-realisation [27].

Academic self-concept reflects a student’s perception of themselves in terms of academic experience and progress [28]. According to Bracken [29], the factors that conform academic self-concept include success and failure to meet expected targets, the perceived difficulty of academic tasks, relationships with other members of the school community (teachers and peers), and self-perception of contribution.

Previous studies have established the relationship between positive self-concept and several interpersonal variables such as higher levels of psychological adjustment and personal competence, a lesser likelihood of behavioural problems and depression, greater resilience against stress, and better academic behaviour [27,30,31].

Few studies have analysed the relationship between our target variables in adolescent populations, but they agree in relating them closely to adaptive behaviours. Extremera, Durán, and Rey [32] point out that students that present adaptive behaviours also show greater levels of vigour in, and commitment to, academic tasks, and Bresó, Schaufeli and Salanova [8] relate both variables to effort, motivation, and abnegation, which are in turn related to higher levels of academic performance. Palacio et al. [33] suggest that high levels of academic engagement and emotional intelligence are closely related to task-oriented behaviour. However, as pointed out by Méndez [34], few studies have related academic self-concept to other variables, and these few are inconclusive, as more academic self-concept-related psychological and academic variables need to be taken into account.

In this context, and given the dearth of works that draw specific links between our target variables, this study aims to analyse the relationship between academic engagement, goal (task)-orientation, and academic self-concept in adolescent students. The research questions are aimed, on the one hand, at the relationship between variables of engagement and academic self-concept, which play a fundamental role in the school environment. On the other hand, we wondered if the goal-task orientation, because it was more self-determined, could mediate the previous relationship between academic engagement and academic self-concept, and what weight it had in this relation on adolescent students.

Two working hypotheses are set forth:

(a) (Task) goal-orientation will be positively related with academic engagement and academic self-concept; and (b) (Task) goal-orientation will perform as a mediating variable in the relationship between academic engagement and academic self-concept in adolescents.

## 2. Method

### 2.1. Sample

This research was composed by 1756 students, both male (N = 914; 52.05%) and female (N = 842; 47.94%), from 12 public secondary schools. Their ages ranged from 12 to 18 years (M = 14.55; SD = 1.68). Participants were selected through two stages: Firstly, the sample was selected through random sampling where all secondary schools had the same probability of being chosen for the research, communicated for it with sufficient advance, and gave their approval in the participation of the research. Secondly, by sample of volunteers who were the students of the educational centres initially selected. Inclusion criteria were the ability to read and communicate in the Spanish language to ensure that they could understand and answer the questionnaire. Incomplete questionnaires were discarded, including those from students who decided to drop out half way and students with cognitive disorders who could not fully understand the questionnaire were excluded from the study. The response rate was approximately 98%. The number of questionnaires discarded amounted to 35, which were not completed in their entirety or were made illegible by the students.

### 2.2. Measurement Scale

Three widely used questionnaires were selected to channel the participant’s responses:

First, the Utrecht Work Engagement Scale—Student (UWES-S) [35] translated into Spanish and validated for use in adolescents [36], was used in order to assess degree of academic engagement. The instrument is comprised of 15 items, divided into three dimensions: vigour (6) (α = 0.75) (e.g., “At my work, I feel bursting with energy”); dedication (5) (α = 0.78) (e.g., “To me, my job is challenging”); and absorption (6) (α = 0.77) (e.g., “I am happy when I am working intensely”). The total score is the average of all three dimensions. Responses range from “Strongly disagree” (1) to “Strongly agree” (5) on a five-point Likert scale. The original questionnaire yielded a Cronbach’s α score of 0.82, and 0.84 in our study.

Goal orientations were measured with Roberts, Treasure and Balagué’s (1998) Perception of Success Questionnaire (POSQ) [37], translated into Spanish and validated by Martínez, Alonso, and Moreno [38]. This questionnaire is comprised of 12 items that reflect the student’s goal orientation, 6 referring to task orientation (e.g., “In class I feel that I am successful when I work hard”), and six to ego orientation (e.g., “In class I feel that I am successful when I show my classmates and teachers that I am the best”). Following our targets and hypotheses, only the first dimension will now be taken into consideration. Responses were given on a 5-point Likert scale ranging from ‘Strongly disagree’ (1) to ‘Strongly agree’ (5). The reliability of this questionnaire in the school environment has been demonstrated in previous studies: Cronbach’s α 0.85 for the task subscale and 0.86 in our study.

Finally, the Academic Self-Concept Scale (ASCS) [39] was used to measure academic self-concept. The instrument is comprised of 13 items divided into two dimensions, academic performance (7) (α = 0.74) (e.g., “I can get good marks”) and academic self-efficacy (6) (α = 0.76) (e.g., “I can carry out the tasks even if they are difficult”). The final result is the average score. Responses range from “Strongly disagree” (1) to “Strongly agree” (5) on a five-point Likert scale. The reliability of the original questionnaire in the school environment has been demonstrated in previous studies: Cronbach’s α 0.75 and 0.79 in our study.

### 2.3. Procedure

The questionnaires were handed out to participants with the cooperation of the schools. Both the students and parents/guardians signed informed consent forms. In coordination with the head of studies, questionnaires were handed out in a single day to all secondary school students in each school where the questionnaire was supplied in each class to all the students attending it. All subjects and parents/guardians were informed of the purpose and nature of the study, thus following the ethical guidelines conveyed by the Declaration of Helsinki [40]. The research protocol was endorsed by the Psychology and Sociology Department, Universidad de Zaragoza. Questionnaires were treated anonymously and participants were voluntary, being allowed to abandon the survey half-way if they wished. For this reason, the questionnaires were provided in each class of the students, not allowing them to take them home.

### 2.4. Data Analysis

Descriptive statistics were used to characterize the social and demographic characteristics of the sample and target variables. Correlations between engagement, (task) goal orientation, and academic self-concept were calculated with IBM SPSS v26.0. Finally, the SPSS v26.0’s MACRO tool was used to carry out mediation analyses by bootstrapping (10,000 runs). For all operations, a *p* ≤ 0.05 level of significance was adopted, with a 95% confidence level.

## 3. Results

### 3.1. Demographics

The sample comprised 1756 students, male (N = 914; 52.05%) and female (N = 842; 47.94%) from 12 public secondary schools. Their ages ranged from 12 to 18 years (M = 14.51; SD = 1.67) (Table 1).

### 3.2. Descriptive Variables

As illustrated in Table 2, male and female students yield similar average results in terms of academic engagement and (task) goal orientation. Males yielded significantly higher scores in terms of academic self-concept, but the differences are not statistically significant.

### 3.3. Correlational Analysis between Academic Engagement, Goal Orientation (Task), and Academic Self-Concept

Correlations between the target variables are presented in Table 3. All the variables correlate with one another, although in different ways. Academic engagement presents a strong positive correlation with (task) goal orientation (0.709 **) and, less clearly, with academic self-concept (0.181 **). (Task) goal orientation is positively correlated with academic self-concept (0.225 **).

### 3.4. Mediation Effects of Goal Orientation (Task) in the Relationship between Engagement and Academic Self-Concept Variables

**Figure 1:**
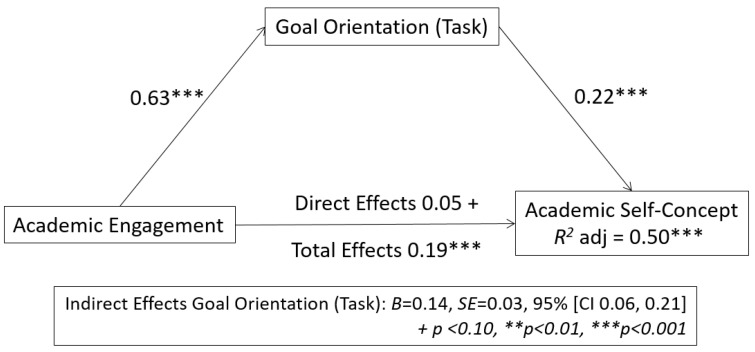
Mediation model of (task) goal orientation in the relationship between academic engagement and academic self-concept variables. + *p* < 0.10, ** *p* < 0.01, *** *p* < 0.001.

In order to assess whether the relationship between engagement and academic self-concept is mediated by (task) goal-orientation, calculations based on Tal-Or, Cohen, Tsarfati, and Gunther [41], using Hayes’s SPSS (v 26.0) [42] Process 3.0 macro (Figure 1) were carried out.

As shown in Figure 1, for (task) it was observed that goal-orientation plays a mediating role in the relationship between engagement and academic self-concept. The results indicate that academic engagement (VI) has an effect on the mediating variable (0.63), and this, in turn, has an effect on academic self-concept (VD) (0.22), (in both cases *p* > 0.001). Zero was not included in the bootstrap interval *B* = 0.14, *SE* = 0.03, 95% [CI 0.06, 0.21] so it can be argued that (task) goal orientation mediates in the relationship between engagement and academic self-concept.

It was observed that academic engagement had no direct positive effect on academic self-concept (0.04, *p* < 0.10) but, when (task) orientation is taken into consideration there is a total effect of 0.18, *p* < 0.001 (direct effect + indirect effect), the proportion of variance being explained by model *R^2^* = 0.50 ***. This suggests that goal orientation plays a mediating role between academic engagement and academic self-concept, a conclusion which has significant practical implications.

In addition, the covariates of gender (*B* = −0.12, *SE* = 0.06, 95% [CI −0.25, 0.13]) and age (*B* = 0.03, *SE* = 0.02, 95% [CI −0.00, 0.07]) were controlled, not being significant in the mediation model.

## 4. Discussion

The aim of this study is to analyse the relationship between academic engagement, (task) goal orientation, and academic self-concept in adolescents.

The first hypothesis stated that (task) goal orientation is positively related to academic engagement and self-concept. According to this hypothesis, task-oriented students will present higher levels of academic engagement and self-concept. The results confirm this hypothesis by showing that goal orientation is positively correlated with academic self-concept, especially academic engagement.

This is generally consistent with the existing literature on goal orientation. On the one hand, numerous studies have shown a clear relationship between (task) goal orientation and academic engagement. Granero, Gómez, Abraldes, and Baena [43] present an adaptive profile characterised by goal orientation and higher degrees of engagement with tasks at hand. Castaño, Rodríguez, and Granero [44] link goal orientation to intrinsic motivations and commitment to school tasks. Bresó, Schaufeli, and Salanova [8] found that self-determined goal orientation is closely related to other variables, such as academic engagement and performance.

On the other hand, other studies establish a direct link between goal orientation and academic self-concept in adolescents. Carranza and Apaza [3] found self-perception and academic achievement to be strongly correlated. García and Musitu [45] present a self-determined profile characterised by intrinsic motivation and task-oriented goal orientation, in relation to higher levels of self-concept, including academic self-concept. For their part, Núñez, Martín, Navarro, and Suárez [46] showed that academic self-concept can be used to effectively predict task-orientated behaviour in adolescents. Finally, Giner, Navas, Holgado, and Soriano [47] found a relationship between task orientation, academic performance, and physical self-concept. All of this confirms the influence of goal orientation on other self-determined variables (including our target variables), especially academic engagement.

The second hypothesis stated that (task) goal orientation plays a mediating role in the relationship between academic engagement and academic self-concept in adolescents. The results also confirm this hypothesis by showing that (task) goal orientation has an effect on both academic engagement and academic self-concept, therefore playing a mediating role between them.

On the one hand, the mediation analysis results show that academic engagement does not have a significant predictive value for academic self-concept. That is, the direct effects of the former on the latter were not statistically significant. On the other hand, if (task) goal orientation is taken into consideration, a significant mediating role between both constructs can be attested. These results show the important role played by (task) goal orientation in adolescents. However, it should be discussed with caution due to the cross-sectional nature of the study where temporal precedence cannot be established. Nonetheless, the mediating role of goal orientation on the other target variables has not been subject to specific studies to date with many present results that can be usefully compared with ours. Fernández, Goñi, Camino, and Zubeldia [48] highlight the fact that school adjustment and adaptive motivational orientations can be used to predict academic self-concept. Other studies establish a direct link between task-orientation and persistence and effort in carrying out school tasks, motivation, and academic self-concept [49], the establishment of good relationships with peers and meeting family academic expectations [50], and academic performance and future aspirations [28]. In this way, some studies suggest that students who are engaged, committed, and integrated in the school community present higher levels of self-concept, are less likely to drop out, and are more likely to believe in the value of effort and abnegation, and thus show adaptive behaviours [34,51,52].

Although, as we have indicated previously, there are not many studies that investigate the specific variables proposed in this research, there are some studies that also approach the study of the relationship between the engagement and academic self-concept constructs in the adolescent student population. Gutiérrez, Tomás, Gómez, and Moll [53] showed the relationship between engagement and academic success with perceptions of task orientations where the climate of mastery and effort in the classrooms play a fundamental role in the learning process of students. Schnitzler, Holzberger, and Seidel [54] maintain the same line of results where it is manifested that those students with the highest level of commitment acquire a beneficial academic self-concept, carrying intrinsic and self-referential motivational implications, in line with our results. Tomás, Gutiérrez, Georgieva, and Hernández [55] highlighted the significant effects of self-efficacy and engagement on the academic achievement of secondary education. In this regard, we must highlight the important role that teachers can play to encourage academic self-concept among students [56], and that students with a high level of academic self-concept are more engaged and committed to school and invest more effort in carrying out school tasks [57].

## 5. Conclusions

These results show that target variables played a significant role in promoting adaptive behaviours characterised by greater levels of academic engagement and a task-oriented goal orientation, which in turn will lead to higher levels of self-concept. This will not only improve their academic performance, but also their personal and psychological development in such an important stage in the life cycle of an adolescence. For prospects, it would be interesting to carry out psychological and academic intervention programs to enhance academic variables as important as student engagement that would strengthen their academic self-concept, keys for proper personal and academic development. At the same time, intrinsic orientations in the assumption of school tasks would help correct the personal and academic development of students. Future studies should examine the relationship between goal orientation and other psychological and academic variables. Similarly, it would also be necessary to take academic performance into consideration, especially in order to examine its relationship with academic self-concept.

The results of our research encourage the scientific community to continue investigating, incorporating new variables, and seeking new questions and research objectives that lead to the personal and academic development of adolescents.

## Figures and Tables

**Table 1:** Results by students’ gender, age, and academic year.

Variables		N	%
**Gender**	Male	914	52.05
	Female	842	47.94
**Age**	12 years	307	17.48
	13 years	293	16.68
	14 years	403	22,94
	15 years	417	23.74
	16 years	269	15.31
	17 years	56	3.18
	18 years	11	0.62
**Academic year**	ESO Year 1	338	19.24
	ESO Year 2	436	24.82
	ESO Year 3	567	32.28
	ESO Year 4	415	23.63

**Table 2:** Results by descriptive variables of academic engagement, goal orientation (task), and academic self-concept.

Variables	Total		Male		Female			
	M	SD	M	SD	M	SD	Cohen’s d	r
Academic Engagement	2.75	1.02	2.76	1.05	2.74	0.98	0.019	0.00
Goal orientation (Task)	2.84	0.90	2.83	0.97	2.86	0.81	−0.033	−0.01
Academic Self-Concept	2.89	1.03	2.95	0.98	2.82	1.08	0.126	0.06

**Table 3:** Results by correlational analysis of academic engagement, goal orientation (task), and academic self-concept variables.

Variables	1	2	3
Academic Engagement	1		
Goal Orientation (Task)	0.709 **	1	
Academic Self-Concept	0.181 **	0.225 **	1

** The correlation is significant at 0.01 (bilateral).
